# Distinctive vasculopathy with systemic involvement due to levamisole long-term therapy: a case report

**DOI:** 10.1186/s13256-018-1728-6

**Published:** 2018-07-16

**Authors:** Bilal Aoun, Mohammad Alali, Jad A. Degheili, Sami Sanjad, Claudine Vaquin, Jean Donadieu, Tim Ulinski, Salah Termos

**Affiliations:** 1 Division of Pediatric Nephrology, Department of Pediatrics, American University of Beirut, Beirut, Lebanon; 2 Hepatobiliary and Transplant Unit, Department of Surgery, Al-Amiri Hospital, Kuwait City, Kuwait; 3 Division of Urology, Department of Surgery, American University of Beirut Medical Center, Beirut, Lebanon; 4 Division of Pediatric Dermatology, Armand Trousseau Hospital, Assistance Publique – Hôpitaux de Paris (AP-HP), Paris, France; 5 Division of Pediatric Hematology, Armand Trousseau hospital (APHP), Paris, France; 6 Division of Pediatric Nephrology, Armand Trousseau Hospital, Assistance Publique – Hôpitaux de Paris (APHP), Paris, France

**Keywords:** Levamisole, Nephrotic syndrome, Cutaneous vasculitis, Neutropenia, Hepatosplenomegaly

## Abstract

**Background:**

Levamisole belongs to the antihelminthic class of drugs that are sometimes administered to patients with frequently relapsing or steroid-dependent nephrotic syndrome, owing to its steroid-sparing effects. Neutropenia and skin lesions, compatible with vasculitis, have been reported as drug complications, but they are rarely associated with any systemic involvement.

**Case presentation:**

We report a case of a 9-year-old Arab boy with steroid-dependent nephrotic syndrome who was treated with levamisole after his third relapse. The drug was initially well tolerated, but mild isolated neutropenia occurred 6 months after levamisole administration. This was followed by cutaneous vasculitis of both ears and the left cheek. The patient also developed hepatosplenomegaly and anemia. Levamisole was discontinued, and his disease remained in remission. All the systemic manifestations disappeared gradually over the course of 1 month. The patient remained in remission until 1 year after levamisole withdrawal, when clinical nephrosis recurred.

**Conclusions:**

Despite levamisole’s being a useful drug for maintaining remission in steroid-dependent nephrotic syndrome, patients on long-term levamisole therapy should be monitored closely to prevent serious complications that can easily be resolved by simple drug withdrawal.

## Background

Levamisole-induced vasculitis is a characteristic cutaneous vasculitic syndrome usually associated with levamisole-adulterated cocaine intake and rarely with the use of levamisole for refractory treatment of nephrotic syndrome [1]. Patients typically present with a painful purpuric rash in a retiform or stellate pattern, with or without central necrosis, involving both extremities, the trunk, nasal tip, digits, cheeks, and ears. Laboratory workup findings may include leukopenia, neutropenia, and positive autoantibodies, mainly antineutrophil cytoplasmic antibodies (ANCAs) [2, 3]. A literature review revealed only a few cases of levamisole-induced vasculitis during treatment of steroid-sensitive and steroid-resistant nephrotic syndrome. The majority of those patients were in the pediatric age group, and their clinical presentation was rarely associated with systemic complications such as persistent neutropenia, hepatosplenomegaly (HSM), and anemia [4]. In this report we describe a distinctive vasculopathy with systemic involvement in a child with steroid-dependent nephrotic syndrome (SDNS) who received long-term levamisole therapy. Complete resolution occurred after drug withdrawal.

## Case presentation

**Fig. 1:**
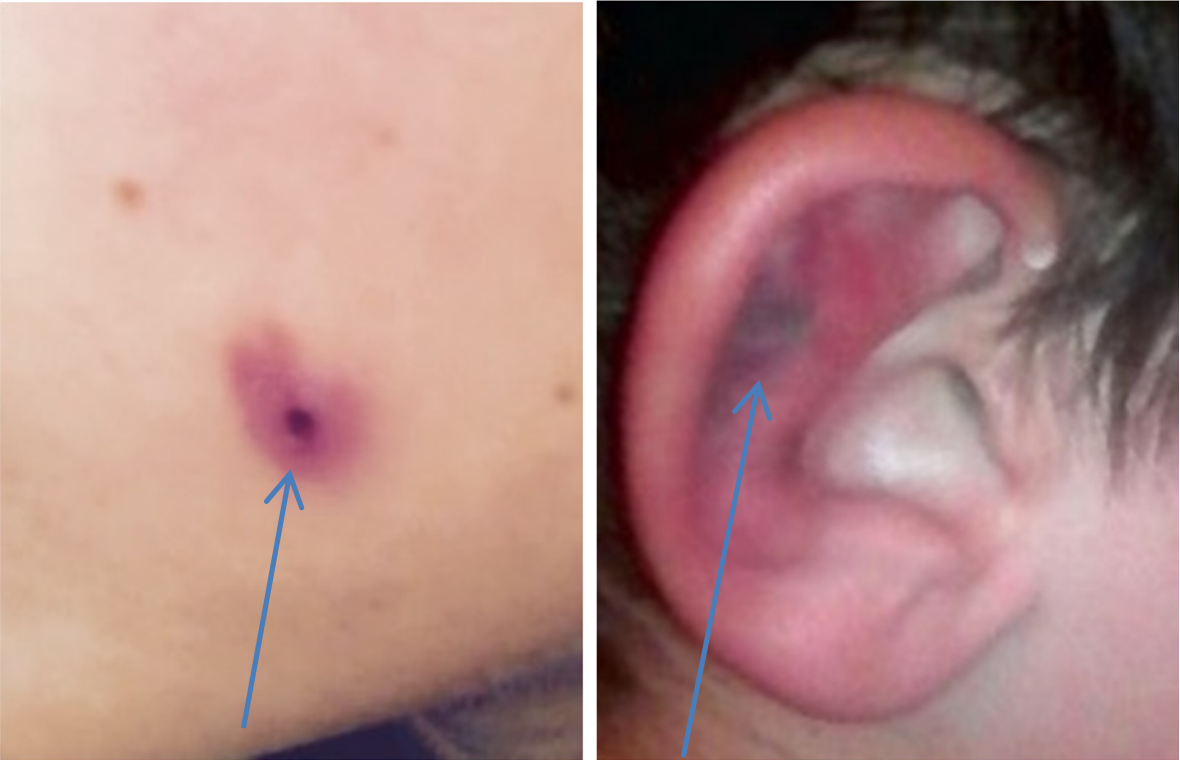
Skin lesions (arrows) present on left cheek and ear pinna, characterized by central necrosis

Our patient was a 9-year-old Arab boy who had had SDNS since the age of 5 years. Because of several relapses over the previous 4 years, and in an effort to spare steroid use and its long-term use complications, the patient was treated with levamisole. His family history revealed that his parents are nonconsanguineous and his father works as a taxi driver. Levamisole was initially well tolerated except for mild isolated and persistent neutropenia (absolute neutrophil count of 1400 cell/mm^3^), which had occurred 6 months after levamisole introduction. The patient had no history of cocaine exposure. Viral infections were ruled out (cytomegalovirus, Epstein-Barr virus, and parvovirus B19). Because the patient’s neutrophil count remained stable and he was in sustained remission, levamisole was maintained at the same dosage (2.5 mg/kg every other day). Six months later, he developed nonspecific lichenoid eruptions on both ears and the left cheek, compatible with cutaneous vasculitis [Fig. 1]. Upon a physical examination, the patient was found to be alert, with vital signs of blood pressure 100/50 mmHg and body temperature 36.7 °C. His heart sounds were regular and rhythmic with a heart rate of 80 beats/min. The result of a neurological examination including sensory and motor responses, especially reflexes, was normal. The boy’s lungs were clear and resonant. His liver and spleen were moderately enlarged. He had lichenoid eruptions on both ears and his left cheek. Ultrasound of the abdomen revealed HSM with liver and spleen lengths of 14 cm and 13 cm, respectively. Mild anemia (hemoglobin 9.7 g/dl) was detected 3 months prior to the appearance of skin lesions. The patient’s kidney function was normal with a creatinine level of 60 μmol/L. Liver function tests reflected by aspartate aminotransferase and alanine aminotransferase showed slightly elevated levels of 120 IU/L (normal range 5–60 IU/L) and 50 IU/L (normal range 7–40 IU/L), respectively, and a normal alkaline phosphatase level of 60 IU/L.

**Fig. 2:**
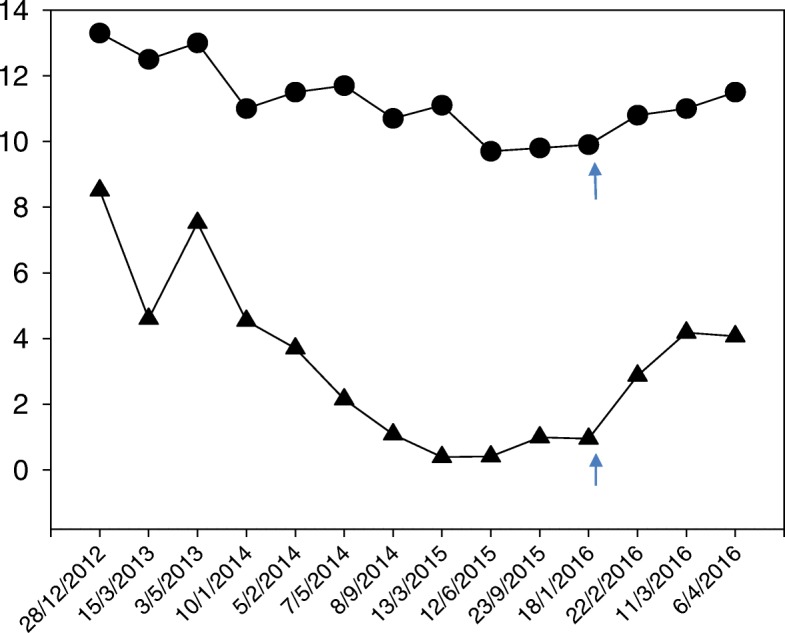
Schematic graph indicating the variations in hemoglobin (Hgb) and neutrophil level (arrows) during period of levamisole administration and withdrawal, along the treatment course. The *dotted line* denotes the Hgb value, and the *arrowhead line* represents the neutrophil count

Immunological investigation revealed an increased positive ANCA count of > 1/640, with negative antinuclear antibodies. Serum complement levels (C3, C4, and CH50) were normal. At this point, levamisole was discontinued. He remained in remission, and his skin lesions disappeared 1 week later. His neutrophil count and hemoglobin levels normalized concomitantly. Furthermore, HSM decreased within 1 month following the withdrawal of levamisole, with liver and spleen lengths becoming 11 cm and 8 cm, respectively. His ANCA levels, however, remained positive (> 1:640) for 12 months after drug cessation. Six months after levamisole withdrawal, the patient remained in remission, with normal white and red blood cell counts and absence of HSM (Fig. 2). One year after levamisole withdrawal, the patient started to develop frequent relapses requiring the combination of steroids and mycophenolate mofetil (MMF) to ensure remission. The patient was never hospitalized and had ambulatory management. Our patient’s clinical course is illustrated in Fig. 2, denoting the relationship between levamisole use, discontinuation, and its marked effect on the neutrophil/hemoglobin levels.

## Discussion

Levamisole was originally marketed as an antihelminthic agent, but it was also found to have major immunomodulatory properties. It induces interferon synthesis and synergizes the effect of steroids and other immunosuppressants. It was used in cancer therapy to treat various immunological renal diseases and skin diseases such as Behçet’s disease. However, the drug was withdrawn from the market in 1999 because of serious side effects, including leukopenia, agranulocytosis, and skin vasculitis [5]. It is still available as a veterinary deworming drug and for treatment of refractory nephrotic syndrome.

Children with SDNS may require several immunosuppressive drugs to control their disease. Such drugs include cyclosporine, cyclophosphamide (CYC), MMF, and levamisole. CYC and MMF have been shown to display long-term efficacy in SDNS [6–8]. As reported previously, levamisole has been considered the least toxic and the least expensive steroid-sparing drug for prevention of relapses in SDNS [9].

Although the exact mechanism of levamisole action in maintaining remission in patients with idiopathic nephrotic syndrome (INS) is still unknown, it is postulated that the drug enhances the Th1-mediated immune response and reciprocally downregulates the Th2 lymphocyte-mediated immune response. Recently, it was suggested that levamisole’s mode of action is attributable to its direct effects on podocytes [10]. On the contrary, the mechanism proposed for drug-induced neutropenia seems to be secondary to antibody formation and autoantibody production, against drug metabolites or protein adducts, covalently attached to neutrophil membrane proteins [11].

INS is a rare disease with an incidence of 2–7 per 100,000 children. It is the most common cause of nephrotic syndrome in the pediatric population [12]. Glucocorticoids remain the treatment of choice in INS to induce remission. However, the disease relapse rate is up to 80%, and many patients may become steroid-dependent in the long term [13] and may require other immunosuppressive agents to maintain remission and limit the corticosteroid’s dosage.

In a study by Bagga *et al.* [14] with 43 children with INS, no significant side effects were experienced; thus, none of the subjects required withdrawal of levamisole. In a case series by Barbano *et al*. [15], a child with INS and prolonged levamisole treatment developed cutaneous vasculitis and HSM, requiring medication withdrawal.

What is unique to our patient is the side effects that he experienced after 6 months of levamisole treatment. Thus, despite levamisole’s being reported as a safe drug, our patient showed that even after a relatively short course of levamisole administration, severe side effects may occur, necessitating its withdrawal.

## Conclusions

Levamisole-induced cutaneous vasculitis with systemic involvement in patients with nephrotic syndrome is a rare complication of a relatively rare disease. Despite its being a useful drug for maintaining remission in patients with SDNS, those maintained on long-term levamisole therapy should be monitored closely, both clinically and by laboratory workup, to detect early abnormalities and prevent serious complications that can be resolved by drug withdrawal.
